# A novel method for linguistic steganography by English translation using attention mechanism and probability distribution theory

**DOI:** 10.1371/journal.pone.0295207

**Published:** 2024-01-02

**Authors:** YiQing Lin, ZhongHua Wang

**Affiliations:** 1 School of Foreign Languages, Xi’an Shiyou University, Xi’an, China; 2 Xi’an Aeronautics Computing Technique Research Institute, AVIC, Xi’an, China; National Textile University, PAKISTAN

## Abstract

To enhance our ability to model long-range semantical dependencies, we introduce a novel approach for linguistic steganography through English translation. This method leverages attention mechanisms and probability distribution theory, known as NMT-stega (Neural Machine Translation-steganography). Specifically, to optimize translation accuracy and make full use of valuable source text information, we employ an attention-based NMT model as our translation technique. To address potential issues related to the degradation of text quality due to secret information embedding, we have devised a dynamic word pick policy based on probability variance. This policy adaptively constructs an alternative set and dynamically adjusts embedding capacity at each time step, guided by variance thresholds. Additionally, we have incorporated prior knowledge into the model by introducing a hyper-parameter that balances the contributions of the source and target text when predicting the embedded words. Extensive ablation experiments and comparative analyses, conducted on a large-scale Chinese-English corpus, validate the effectiveness of the proposed method across several critical aspects, including embedding rate, text quality, anti-steganography, and semantical distance. Notably, our numerical results demonstrate that the NMT-stega method outperforms alternative approaches in anti-steganography tasks, achieving the highest scores in two steganalysis models, NFZ-WDA (with score of 53) and LS-CNN (with score of 56.4). This underscores the superiority of NMT-stega in the anti-steganography attack task. Furthermore, even when generating longer sentences, with average lengths reaching 47 words, our method maintains strong semantical relationships, as evidenced by a semantic distance of 87.916. Moreover, we evaluate the proposed method using two metrics, Bilingual Evaluation Understudy and Perplexity, and achieve impressive scores of 42.103 and 23.592, respectively, highlighting its exceptional performance in the machine translation task.

## 1. Introduction

Linguistic steganography refers to the technique of concealing secret information within text, making it difficult for people to perceive its presence. Unlike other forms of steganography, linguistic steganography focuses on utilizing language and semantical features to hide information [1]. In the field of linguistic steganography, information can be concealed by altering word order, using specific grammar structures, or incorporating unusual vocabulary in the text. The hidden information may include encrypted messages, secret instructions, or any other content that needs to be kept confidential [2]. The applications of linguistic steganography are diverse and have far-reaching implications in the fields of information security and network security. Some of these applications include:

1. Covert Communication: Linguistic steganography can be used to covertly exchange sensitive information between parties. By concealing data within seemingly innocuous text, it offers a level of discretion that can be vital in various situations, including intelligence operations and secure corporate communications.
2. Privacy Preservation: Linguistic steganography can be a means of preserving the privacy of communications. In cases where individuals or organizations need to protect their data from unauthorized access or surveillance, this technique allows for discreet information exchange.
3. Censorship Evasion: In regions with strict censorship policies, linguistic steganography can serve as a way to bypass content restrictions. By hiding information within seemingly innocuous text, individuals can share and access information that would otherwise be prohibited.
4. Secure Communication: Linguistic steganography can facilitate secure communication by embedding encrypted messages within carrier text. This method enhances the confidentiality of the information being transmitted, making it harder for eavesdroppers to intercept and understand the content.
5. Security Protocols: Some security protocols and systems utilize linguistic steganography to embed digital watermarks or additional security features within textual documents to prevent forgery or unauthorized access.

Linguistic steganography offers a unique and versatile approach to data security, providing a covert channel for the exchange of sensitive information, all while maintaining the appearance of regular text. As technology continues to advance, the development and analysis of linguistic steganography techniques play a crucial role in ensuring the confidentiality and integrity of digital communications [3].

In the early stages of linguistic steganography, modification-based techniques were commonly used, such as synonym substitution, introducing spelling errors, syntactic transformations, and semantical operations, to embed secret information [4, 5]. However, these methods heavily relied on complex syntactic or semantical analysis, making it challenging to achieve high accuracy [6]. In addition, attackers could potentially detect the modifications through comparisons, resulting in lower security [7]. Moreover, due to the limited redundancy in the text, these techniques had a smaller embedding capacity, and even minor text alterations could lead to semantical anomalies or grammatical errors.

In response to these issues, researchers have proposed non-modification-based steganographic methods, where carrier texts are obtained or generated under the guidance of secret information [8]. These carrier-based methods aim to find a series of texts that match the secret information, for instance, by selecting several texts from a large corpus using the mapping function. On the other hand, generative linguistic steganography relies on specific statistical patterns or language models to automatically produce steganographic texts [9]. Early generative steganography often relied on grammar rules, such as context-free grammars or sentence templates. TEXTTO was one of the earliest methods [10], designed with sentence templates composed of word groups. Based on the syntactic features of the sentences, these templates were filled to generate the carrier texts. Other early studies, like NICETEXT [11] and Mimicry [12], utilized grammar rules to generate carrier texts. The key difference of these methods from modification-based linguistic steganography is that they do not require the original texts, making it difficult for attackers to detect by comparison. Although these methods have a high embedding rate, they lack consideration of semantical information, resulting in carrier texts with no contextual relevance, leading to lower security.

To address the issue of semantical irrelevance, some generative steganography models using statistical language models emerged, such as using n-gram models [13] or Markov chains [14] to model semantical features. Due to the difficulty of semantical modeling, some statistical models are applied to specific genres such as short jokes [15], emails [16], and poetry [17]. To improve text fluency, Guo et al. [18] used n-gram models to generate alternative carrier text, which was then manually edited and polished. This method calculates the conditional probability *p*(*x*_*i*_|*x*_1_, *x*_2_,…, *x*_*i*-1_), where *x*_*i*_ is the *i*-th word, to determine the word at each moment. To address data sparsity and excessive parameters, both n-gram models and Markov models introduce the Markov assumption, assuming that each word is only related to the previous several words, i.e., *p*(*x*_*i*_|*x*_1_, *x*_2_,…, *x*_*i*-1_) ≈ *p*(*x*_*i*_|*x*_*i-n*+1_, *x*_*i-n+*2_,…, *x*_*i-*1_). However, according to the Markov assumption, as the word distance increases, the semantical relevance between words decreases, resulting in weak semantical relevance between sentences in the entire text [19].

In recent years, neural networks have provided new solutions for modeling long-range semantical dependencies in text. Sun et al. [20] combined the Encoder-Decoder architecture of RNN (Recurrent Neural Network) with grammatical templates to generate hidden Chinese poetry, improving the long-range semantical relevance between generated carrier text. Cao et al. [21] used Long Short-Term Memory network (LSTM) to select encoded carrier words from a specific word library that matches the secret message. Yang et al. [22] proposed an RNN-Stega algorithm for information hiding, achieving state-of-the-art performance in embedding capacity and text quality. The algorithm first encodes alternative words based on conditional probability distributions and then picks words that match the current secret message bitstream for embedding. As traditional models have limited long-term memory compared to RNN and LSTM, deep learning-based models have gradually replaced traditional linguistic steganography models [23]. However, RNN memory units also have certain limitations: As the vocabulary grows, previous semantical information is eventually ignored, resulting in weaker semantical consistency in the entire text [24]. Therefore, how to maintain semantical relevance when generating long or multiple sentences of carrier text remains a challenge in linguistic steganography.

This paper combines the neural machine translation (NMT) model with steganography models to introduce the NMT-Stega model. The proposed NMT-Stega model generates the carrier text y of target language based on the source text *x* and previously generated target words. During text generation, the source text provides useful information for semantical relevance between sentences. For example, when generating the word *y*_*n*+1_, NMT-Stega considers not only the previously generated words *y*_1_, *y*_2_,…, yn but also the corresponding source words *x*_1_, *x*_2_,…, *x*_*m*_. Furthermore, the model uses an attention-based hyper-parameter to equilibrate the impact of the source text and the target text on the target word. In order to further enhance the quality of hidden text, this paper introduces a word pick policy to construct an alternative set. Experimental results demonstrate that NMT-Stega has the capability to generate multiple semantically related lengthy sentences. Additionally, NMT-Stega exhibits excellent performance in anti-steganography experiments.

The following three aspects are where our contributions lie:

1. Proposed NMT-Stega method addresses the issue of decreased semantical relevance as the length of generated sentences increases. It adopts an Encoder-Decoder architecture fused with attention mechanism to dynamically embed the secret information during text generation. Each target word’s pick takes into consideration both the source text and previously generated target text to maintain semantical coherence with distant words.
2. To mitigate the degradation of text quality caused by embedding secret information, a dynamic word pick policy based on probability variance is designed, allowing adaptive construction of the alternative set and dynamic adjustment of embedding capacity at each time step.
3. Attention hyper-parameters are introduced to study their effects on the embedding capacity and text quality, providing insights into the interplay between different attention parameters and variance thresholds.
4. The article makes a significant contribution by proposing a novel method that combines attention mechanisms and probability theory for linguistic steganography. This approach could potentially improve the security and quality of steganographic text, which is a valuable contribution to the field.

## 2. Materials and methods

### 2.1 Background

#### 2.1.1 Linguistic steganography by machine translation

Translation-based Steganography (TBS) is a technique that leverages the variability of different translations produced by multiple translators for the same source text. Initially proposed by Beltrán et al. [25], the Lost in Translation (LiT) model utilizes this characteristic to hide information within the translated sentences. Each translator’s output is encoded using Huffman coding, and the sentences that correspond to the encoding of the secret message bits are selected as the final carrier texts.

An improved version of LiT, known as LiJtT (Lost in just the Translation), was later introduced by Zidenberg et al. [26]. Unlike LiT, LiJtT directly encodes the generated translated sentences. Each sentence is transformed into a hash value based on a secret key, and the sentences that match the least significant bit (LSB) of the secret message bits are chosen as the carrier texts.

Both LiT and LiJtT require the involvement of multiple translators, and the embedding capacity of the model depends on the diversity of the generated translations [27]. However, if there are no LSB(Least Significant Bit) matches with the secret message bits in any translation at a given moment, the embedding process fails. To address this issue, Meng et al. [28] proposed LinL (Lost in *n*-best List), which utilizes a statistical machine translation (SMT) model to obtain the n-best translations for a given source sentence.

For example, when *n* = 1, given a source sentence, the translated sentence $\hat{t}$ is the sentence that maximizes the conditional probability *p*(*t|s*), i.e.:

$$\hat{t}=\underset{t}{\text{arg}\;\text{max}\;p(t|s)}$$ (1)

According to Bayes’ formula:

$$\hat{t}=\underset{t}{\text{arg}\;\text{max}\;p(t)p(s|t)}$$ (2)

In which, *p*(*t*) represents the language model of the target text, and *p*(*s|t*) represents the translation model trained with a bilingual corpus.

LinL employs an n-best search algorithm to select the n-best translated sentences and encodes them for information embedding. Compared to LiT and LiJtT, LinL demonstrates improved robustness and higher embedding capacity.

Existing TBS models are based on SMT models. One problem with SMT is that it uses the Markov assumption to compute the language model, which assumes that the generation of the next word depends only on a few preceding words. As a result, the quality of the generated translation text is relatively poor [19].

#### 2.1.2 NMT

**Fig 1:**
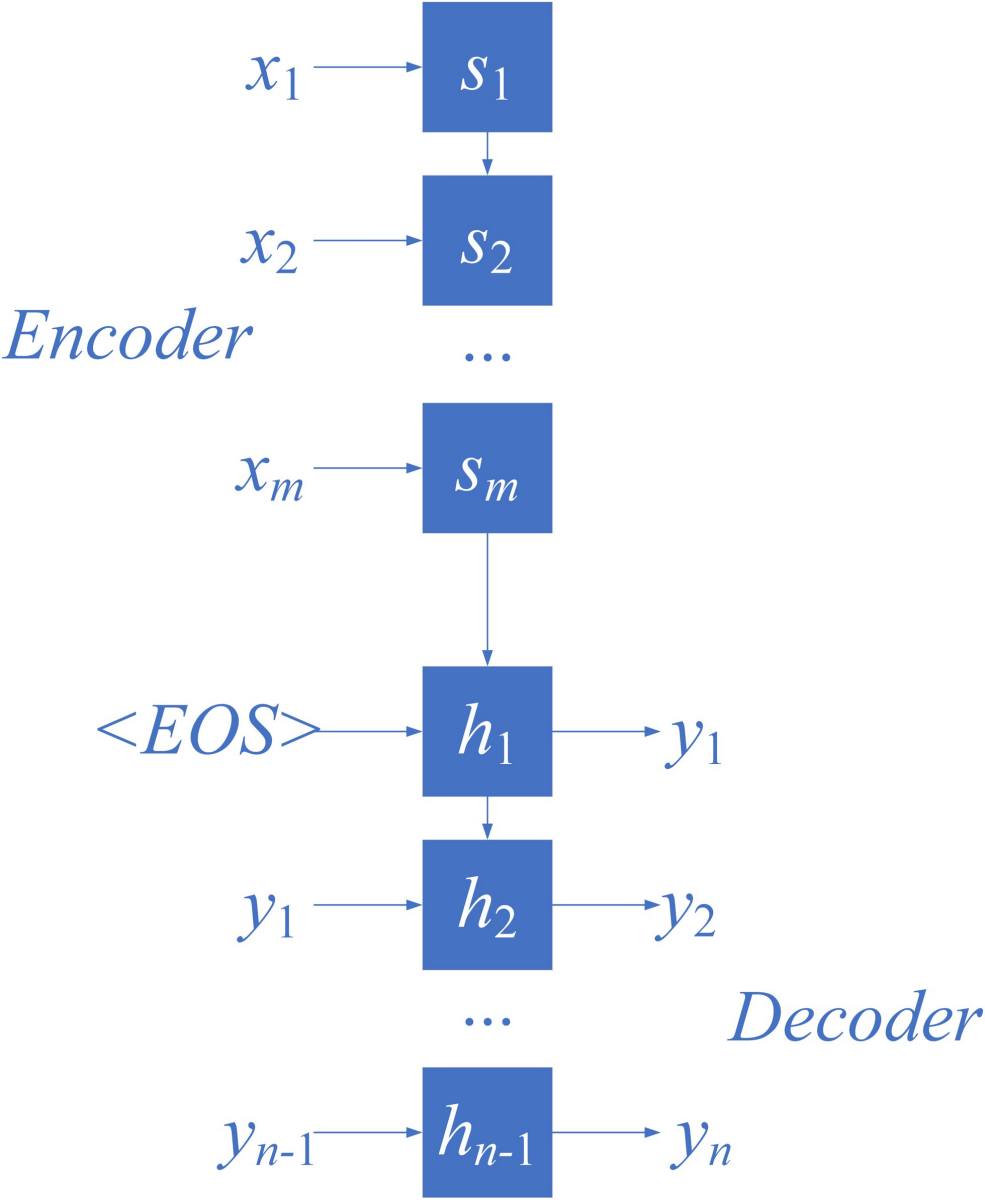
NMT model.

Recently, NMT has demonstrated outstanding performance in various machine translation tasks such as English-German and English-French translations [29, 30]. Unlike SMT, NMT consists of an Encoder-Decoder architecture. The Encoder encodes the source sentence into a fixed-length vector, which is then fed into the Decoder to generate the translation in the target language [23], as shown in Fig 1.

Let *x* = (*x*_1_, *x*_2_,…, *x*_*m*_) represent the current input source sentence to the Encoder, where m is the number of words in *x*. Let *y* = (*y*_1_, *y*_2_,…, *y*_*n*_) be the final output target sentence from the Decoder, where *n* is the number of words in *y*. At time step *t*, the NMT model predicts the target word *y*_*t*_ by calculating the conditional probability as follows:

$$p\left(y_{t}|y_{1},y_{2},\ldots ,y_{t}{}_{-1},x\right)=\text{softmax}\left(g\left(h_{t}\right)\right)$$ (3)

Here, ht is the recursive hidden state computed according to (4), and g(·) is the transformation function that converts ht into a word vector, with the word vector’s dimension equal to the size of the vocabulary.

$$\begin{array}{l} h_{t}=f\left(h_{t-}{}_{1},y_{t-}{}_{1}\right),t\geq 2\\ \text{s.t}.\;h_{1}=f\left(c,y_{0}\right),t=1 \end{array}$$ (4)

Where *c* is the representation of the source sentence obtained from the Encoder, and *f*(·) is a non-linear function, which can be an RNN, LSTM, GRU (Gate Recurrent Unit), or Transformer. Each sentence is represented with a start symbol <*SOS*> and an end symbol <*EOS*>, with *y*_0_ = <SOS>.

Due to NMT’s superior performance across translation tasks compared to SMT, it has gained significant concern. The attention mechanism enables the model to focus on specific words in the source sentence when generating target words, significantly improving the quality of the generated translation text [31], this is also one of the sources of inspiration for this paper.

### 2.2 Proposed method

In order to obtain high-quality steganographic text with inter-sentence semantical correlation, this paper proposes a NMT-based steganographic model called NMT-Stega. To fully leverage useful information from the source text during the generation process, an attention mechanism is employed. Traditional TBS method based on SMT generates multiple translation sentences from the source sentence, encodes each sentence, and finally selects the one corresponding to the secret information as the final target sentence. In contrast, NMT-Stega dynamically selects generated words based on the secret information during the target sentence generation process, achieving the goal of embedding secret information.

Since the selected words are not always the most probable ones, embedding process may lead to a decrease in the quality of the generated text to some extent. The main focus of this paper is to maintain inter-sentence semantics during the information embedding process while minimizing the quality degradation caused by word pick.

#### 2.2.1 General architecture

To improve translation accuracy, this paper adopts an NMT model based on attention mechanism as the translation model. Integrating attention mechanism into the Encoder-Decoder structure allows the model to dynamically focus on specific parts of the input, thereby enhancing the efficiency of natural language processing (NLP) tasks. For instance, in machine translation, the attention mechanism can find highly relevant source language words when predicting the output value y_t_.

**Fig 2:**
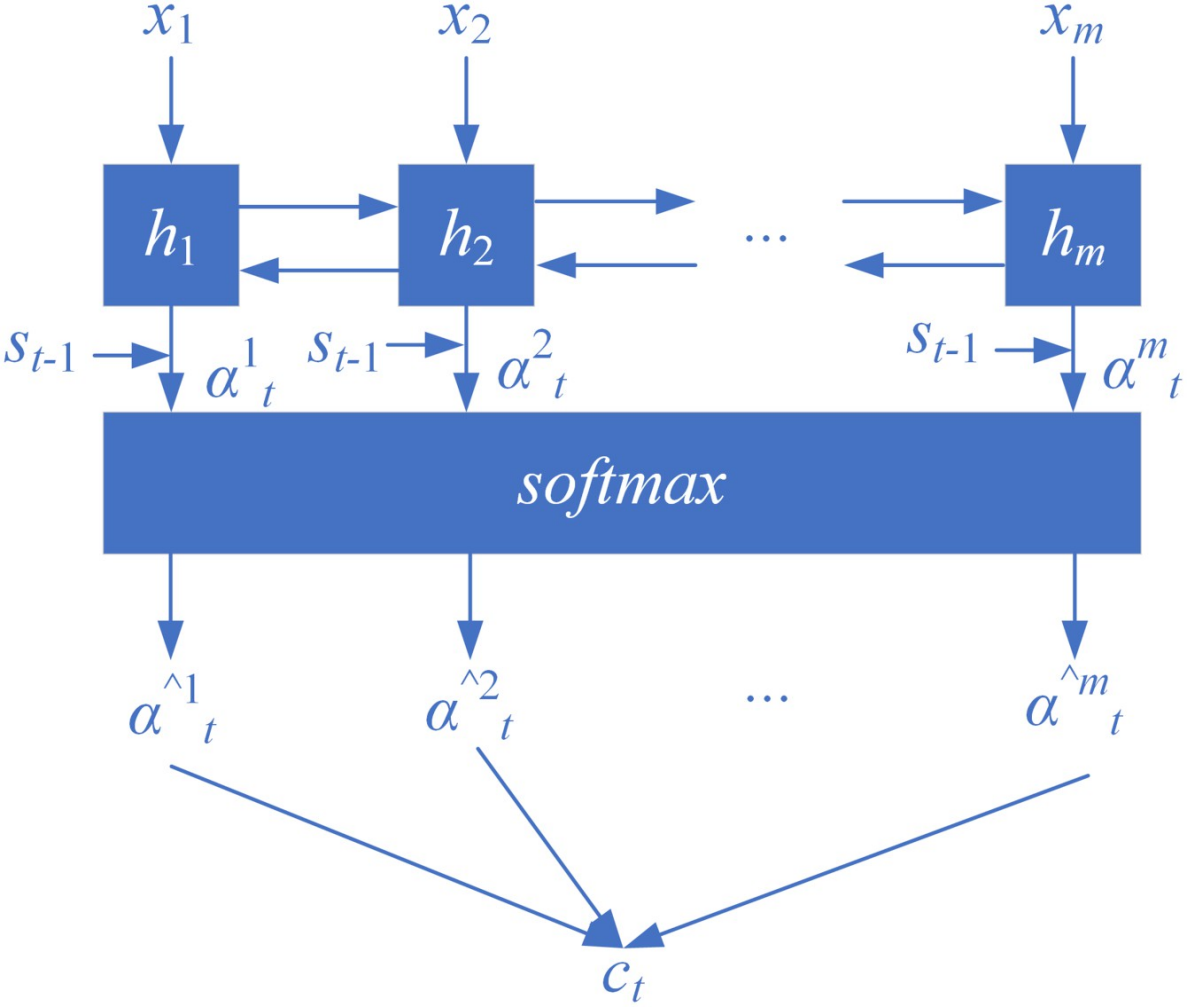
Encoder of NMT-Stega model.

**Fig 3:**
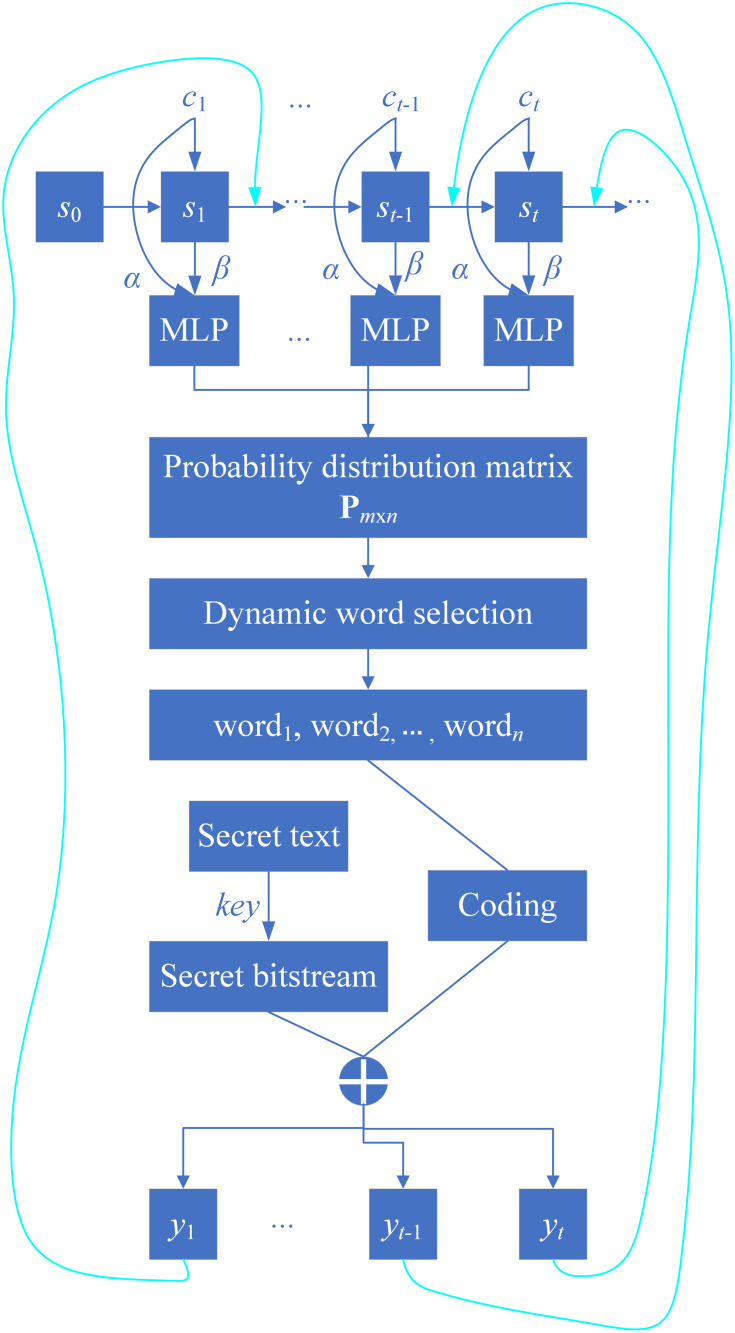
Decoder of NMT-Stega model.

The architecture of the proposed NMT-Stega model is illustrated in Figs 2 and 3. Next, the working principles and roles of the encoder and decoder will be introduced separately.

Let $\vec{x}=\;(x_{1},\;x_{2},\;\ldots ,\;x_{m})$ represent the current input sentence to the encoder, where m is the number of words in $\vec{x}$. The hidden layer *h* can take the form of RNN, LSTM, or GRU. In this paper, bidirectional LSTM (Bi-LSTM) is used as the hidden unit, which can better capture contextual information within sentences, as it compresses not only the information preceding the current word but also the information following it. In other words, each word *x*_*t*_ can be represented as *h*_*t*_, which is a fusion of the forward hidden state ${\vec{h}}_{t}$ and the backward hidden state ${\overset{\leftarrow }{h}}_{t}$:

$${\vec{h}}_{t}=f_{LSTM}(x_{t},{\vec{h}}_{t-1})$$ (5)

$${\overset{\leftarrow }{h}}_{t}=f_{LSTM}(x_{t},{\overset{\leftarrow }{h}}_{t+1})$$ (6)

$$h_{t}={\left[{\vec{h}}_{t}^{T};{\overset{\leftarrow }{h}}_{t}^{T}\right]}^{T}$$ (7)

The decoder’s final output is *y* = (*y*_1_, *y*_2_,…, *y*_*n*_), where n is the number of words in the target sentence *y*. The probability distribution for predicting the current word *y*_*t*_ can be represented as:

$$p\left(y_{t}|y_{1},\;y_{2},\;\ldots ,\;y_{t-1},\;\vec{x}\right)=MLP(y_{t-1},\;s_{t},\;c_{t})$$ (8)

where MLP is a multi-layer perceptron component, st is the output state of the hidden layer, which is given by:

$$s_{t}=f_{LSTM}(s_{t-1},y_{t-1},c_{t})$$ (9)

where, ct is another hidden state besides *h*_*t*_, computed as follows:

$$c_{t}=\sum_{i=1}^{m}{\hat{\alpha }}_{t}^{i}h_{i}$$ (10)

where ${\hat{\alpha }}_{t}^{i}$ is the attention weight, calculated as follows:

$${\hat{\alpha }}_{t}^{i}=\text{softmax}(\alpha_{t}^{i})$$ (11)

Where $\alpha_{t}^{i}$ represents the reference weight of the current word *y*_*t*_ on the source word *x*_*i*_, given by:

$$\alpha_{t}^{i}=a(s_{t-1},h_{t})$$ (12)

The component a is a feedforward neural network trained together with the other parts of the NMT model.

#### 2.2.2 Dynamic word pick policy

Applying the proposed word pick policy to the obtained probability distribution allows us to generate an alternative set, and the effectiveness of the word pick policy directly determines the quality of elements in the alternative set. In this experiment, we determine whether a word can enter the alternative set by setting a limit on the variance of the probability distribution.

First, we need to select the top-8 words with the highest probabilities from the obtained probability distribution. The probability value ranked first is denoted as *p*_1_, corresponding to the target word *target_word*_1_ generated at time t in the case of no embedding. We then calculate the variance values var(n) (n = 2, 4, 8) for the top-8 words, specifically:

$$var(n)=\frac{1}{n}\sum_{i=1}^{n}{\left(p_{i}-\frac{1}{n}\sum_{j=1}^{n}p_{j}\right)}^{2}<\varepsilon$$ (13)

where pi represents the probability value of the *i*-th word *w*_*i*_ at time *t*, sorted in descending order of probability, and ε is the variance threshold. If the variance of the top-8 words’ probabilities satisfies the threshold condition, i.e., var(8) is less than *ε*, then all 8 words are added to the alternative set. Otherwise, we reduce n to 4 and check if var(4) is less than ε. If it satisfies the condition, we add the first 4 words to the alternative set. If not, we further reduce n to 2 and check if var(2) is less than ε. If it satisfies the condition, the current alternative set consists of 2 words. If not, we skip the embedding at the current position and directly output the word with the highest probability.

Different values of *ε* will produce different alternative sets. The impact of *ε* on the model will be detailed in the experimental section.

The proposed word pick policy allows the number of words in the alternative set at each time step to be potentially different. In other words, the embedding capacity at each time step is automatically adjusted based on the current conditional probability distribution, making it an adaptive secret information embedding policy.

#### 2.2.3. Our attention-based hyper-parameter adjustment

In our model, the probability distribution of the target word at time *t* depends on both the source text and the already generated target text. The calculation formula is as follows:

$$p\left(y_{t}|y_{1},\;y_{2},\;\ldots ,\;y_{t-1};x_{1},\;x_{2},\;\ldots ,\;x_{n}\right)=MLP(\alpha \cdot C_{t},\;S_{t})$$ (14)

Where, *α* is the attention hyper-parameter, and it satisfies *α*∈[0, 1]. MLP represents a multi-layer perceptron. During model training, *α* is set to 1. During generation, α is adjusted to control the degree of dependency between the target sentence and the source sentence. When *α* = 0, the generation of the word at time *t* only depends on the already generated target sentence, which represents the traditional generative linguistic steganography method. Due to the lack of dependency on the source text, the generated target sentences lack semantical relevance. As *α* increases, the embedding of the secret word at time *t* gradually depends on the source text. When *α* = 1, the dependency of the secret word on both the source and target sentences becomes consistent.

In this paper, we manually pick different attention parameter weights to test the model’s text generation quality and steganography ability. Different weights will yield different probability distributions, thereby changing the word pick in the alternative set.

Embedding and extraction algorithms. The main idea of the embedding algorithm is to construct a alternative set based on the probability distribution generated by the language model and then select the word corresponding to the current moment’s secret information as the final embedded word. The specific embedding process is shown in **Algorithm 1**.

**Algorithm 1. Secret information embedding algorithm**.

**Input**: Secret information bitstream *B*; Source text *C*; Beam size *bs*; Variance threshold *ε*; Weights *α*, *β*.

**Output**: Target embedded word.

**Procedure**:

Step 1: Data preprocessing and model training.

Step 2: While *B* is not empty do:

Step 3: Read a sentence from the source text *C*.

Step 4: If not at the end of the sentence then:

Step 5: Calculate the probability distribution of the next word using the model according to (14).

Step 6: End if.

Step 7: For *bs* = 8; *bs* > 0, do:

Step 8: If *var*(*bs*) < ε then:

Step 9: Add these *bs* words to the alternative set.

Step 10: Else:

Step 11: *bs* = *bs*-2.

Step 12: End if.

Step 13: End for.

Step 14: Build a binary tree based on the probability distribution of alternative words in the alternative set and encode the alternative words.

Step 15: Generate the target word corresponding to the binary code that matches the current secret information bit.

Step 16: End while.

Step 17: Return the generated target embedded word.

For example, assuming a secret bit stream B = {01011011…}, and the source text is *He said he hopes that the two sides will further strengthen their exchanges and cooperation* (In Chinese). The alternative set size is 2, and the current secret bit is 0. Then, we select the word with the highest probability as the embedded word. If the next bit is 1, we choose the word with the second-highest probability as the embedded word. Based on the secret bit stream, the final embedded sentence obtained will be *He said he hopes that the two sides will further strengthen their exchanges and cooperation* (In English).

The process of secret information extraction is similar to embedding. The receiver shares the source text and the same NMT model with the sender. Then, the same method is used to build the alternative set, encode alternative words, and compare the received stego-text with alternative words to extract the corresponding secret information bits. The specific extraction algorithm is shown in **Algorithm 2**.

**Algorithm 2. Secret information extraction algorithm**.

**Input**: Target carrier text; Source text C; Beam size bs; Variance threshold ε; Weights α, β.

**Output**: Secret information bitstream *B*.

**Procedure**:

Step 1: Data preprocessing and model training.

Step 2: Read a sentence from the source text *C*.

Step 3: If not at the end of the sentence then:

Step 4: Calculate the probability distribution of the next word using the model according to (14).

Step 5: End if.

Step 6: For *bs* = 8; *bs* > 0, do:

Step 7: If *var*(*bs*) < ε then:

Step 8: Add these *bs* words to the alternative set.

Step 9: Else:

Step 10: *bs* = *bs*-2.

Step 11: End if.

Step 12: End for.

Step 13: Build a binary tree based on the probability distribution of alternative words in the alternative set and encode alternative words.

Step 14: Compare the received hidden sentence with alternative words, extract the corresponding secret information bits.

Step 15: Add the extracted bits to *B*.

Step 16: Return the secret information bitstream *B*.

## 3. Experiments and results

This paper conducted a series of experiments to test the embedding rate, text quality, and security of the generated carrier text. In addition, a comparison with other generative linguistic steganography models was performed to assess the model’s performance in preserving text semantics.

### 3.1 Data acquisition, processing and experimental setup

The proposed model used a parallel Chinese-English corpus obtained from the public news website as the dataset, comprising 1,252,977 news sentences. The maximum and average sentence lengths were 98 and 34, respectively. The dataset was split into training, validation, and testing sets with an 8:1:1 ratio. Before model training, data preprocessing was performed, including removing special symbols, website links, and numerical characters.

All test experiments in this paper were carried out on the Ubuntu 18.4 operating system with a GX2080Ti GPU (128GB) and CUDA 10.0. The model was implemented using Pytorch and Python 3.8. The hyper-parameters of the model were set as follows: encoder and decoder with 6 stacked layers, each layer using an 8-head attention mechanism; word embedding dimension set to 512; dropout regularization (dropout rate = 0.2) during pretraining to avoid overfitting; Adam optimization algorithm with an initial learning rate of 0.0003. Additionally, a batch size of 64 and 75 iterations were used.

**Fig 4:**
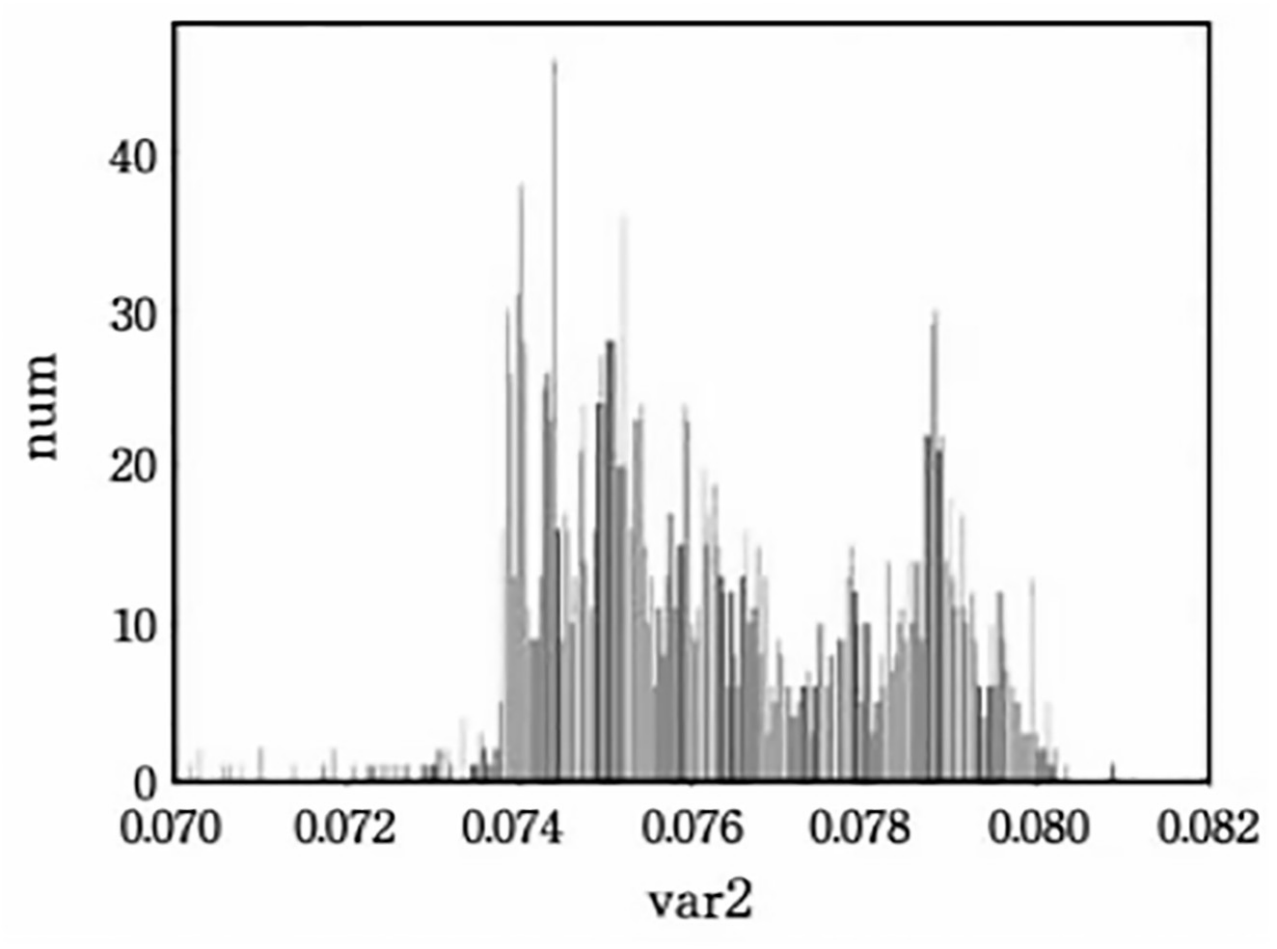
The conditional probability variance histogram of top-2.

**Fig 5:**
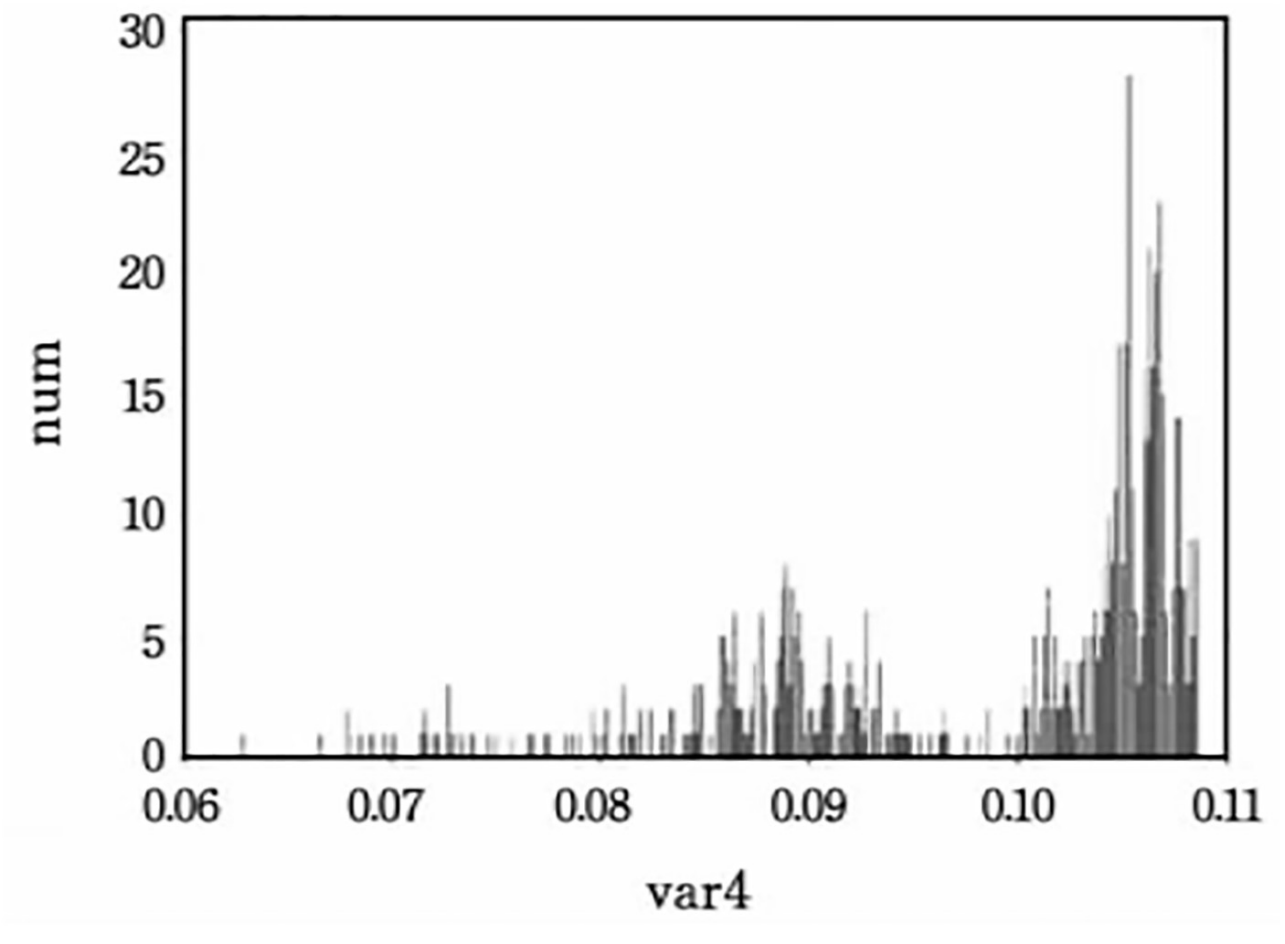
The conditional probability variance histogram of top-4.

**Fig 6:**
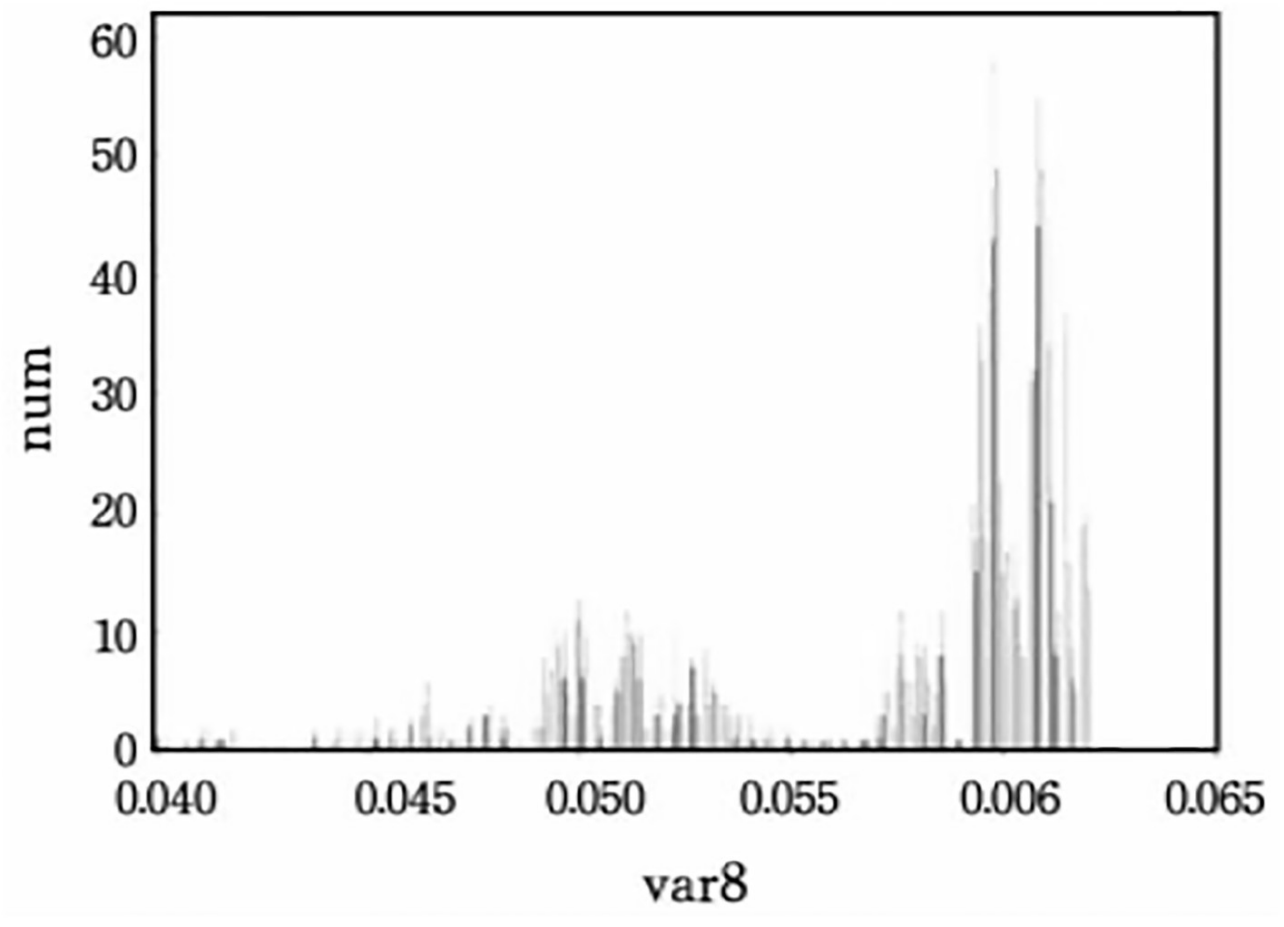
The conditional probability variance histogram of top-8.

To ensure the quality of the generated carrier text, setting a reasonable variance threshold is essential. This paper first calculated the variance distributions of the top-2, 4, and 8 words without embedding, and their corresponding histograms are shown in Figs 4–6.

Based on the obtained histograms, manual threshold values were selected: 0.07, 0.072, 0.074, 0.076 for the top-2; 0.07, 0.08, 0.09, 0.1 for the top-4; 0.045, 0.05, 0.055, 0.06 for the top-8. The impact of variance thresholds on evaluation metrics will be further demonstrated in the following experiments.

### 3.2 Evaluation metrics

In machine translation systems, BLEU (Bilingual Evaluation Understudy) [32] and PPL (Perplexity) [33] are commonly used to evaluate text quality. BLEU is a metric that measures the similarity between machine translation and professional translation. Higher *BLEU* values indicate higher translation quality, and its calculation is as follows:

$$BLEU_{N}=b(C,S)\;\text{exp}(\sum_{n=1}^{N}w_{n}\;\text{log}\;CP_{n}(C,S))$$ (15)

Where *b*(*C*, *S*) is a penalty factor:

$$b(C,S)=\left\{\begin{array}{l} 1,l_{s}\leq l_{c}\\ e^{1-\frac{l_{s}}{l_{c}}},l_{c}\leq l_{s} \end{array}\right.$$ (16)

Where, *l*_*s*_ is the length of the reference sentence, and *l*_*c*_ is the length of the evaluated sentence. *CP*_*n*_(*C*,*S*) represents the precision of generated translation *C* compared to reference translation *S*:

$$CP_{n}(C,S)=\frac{\sum_{i}\sum_{k}\;\text{min}\;(h_{k}(c_{i}),\;\text{max}\;h_{k}(s_{ij}))}{\sum_{i}\sum_{k}h_{k}(c_{i})}$$ (17)

PPL is a metric used to evaluate the quality of language model. It treats the language model as a probability distribution over sentences or paragraphs, representing the probability of generating a sentence in the text. A smaller *PPL* indicates a better-trained model, and its calculation is as follows:

$$PPL=2^{-\frac{1}{N}\sum_{i=1}^{N}\text{log}\;P(s_{i})}$$ (18)

Where *s*_*i*_ represents the *i*-th generated sentence, *N* is the total number of sentences, and *P*(*s*_*i*_) is the probability of *s*_*i*_ calculated by the language model. In this paper, BLEU was used to assess text quality, while PPL was used to evaluate the statistical performance of the generated text.

### 3.3 Ablation experiment

Table I presents example target texts generated with different values of the attention hyper-parameter *α*.

From Table 1, it can be observed that sentences generated under different attention hyper-parameters are different, but they share similar semantical attributes. Therefore, further research on the relationship between attention hyper-parameters and model performance is necessary. This section will discuss the impact of different *α* values on the model’s embedding rate and the quality of the generated text, using *bs* = 2 as an example.

**Table 1:** Example target texts generated with different attention HYPER-parameter.

(A)	
*α*	Translation
1	It is not the level and number of people that we have seen.
0.9	There has been a long time, but not the level and number of people we have seen now.
0.8	There is always a problem, but not the level and numbers that we have seen now.
0.7	However, it is not the case that we have seen in the level and number of people.
0.6	It is not enough to see that the current level of work.
(B)	
*α*	Translation
1	During the passage of typhoon disaster, we found in the sky and some trees on the ground were found damaged by road safety.
0.9	During the passage of typhoon, certain trees on the slope were posed as a safety hazard in August last year.
0.8	During the passage of typhoon, some trees on the slope were once burning and posed a risk of fallen trees.
0.7	During the passage of typhoon course, there were many trees on the slope of Sheungyiu last August and posed a challenge to road safety.
0.6	During the course of typhoon, it was forbidden to grow down on the ground and posed a chance to prevent the spread of land.
(C)	
*α*	Translation
1	The best way is to encourage enterprises of the two countries to explore areas and content of cooperation, and the government has given positive support.
0.9	The best way is to encourage the companies of both sides to explore areas and content of cooperation, and the government should give positive support.
0.8	The best way is to encourage the enterprises of the two countries to explore areas and content of cooperation, and to offer positive support.
0.7	The best way to promote cooperation is to encourage enterprises of the two sides to explore new ways to expand cooperation and to give them positive support.
0.6	The best way to promote cooperation is to encourage enterprises to discuss ways to expand cooperation.

#### 3.3.1 The influence of α on the embedding rate

**Fig 7:**
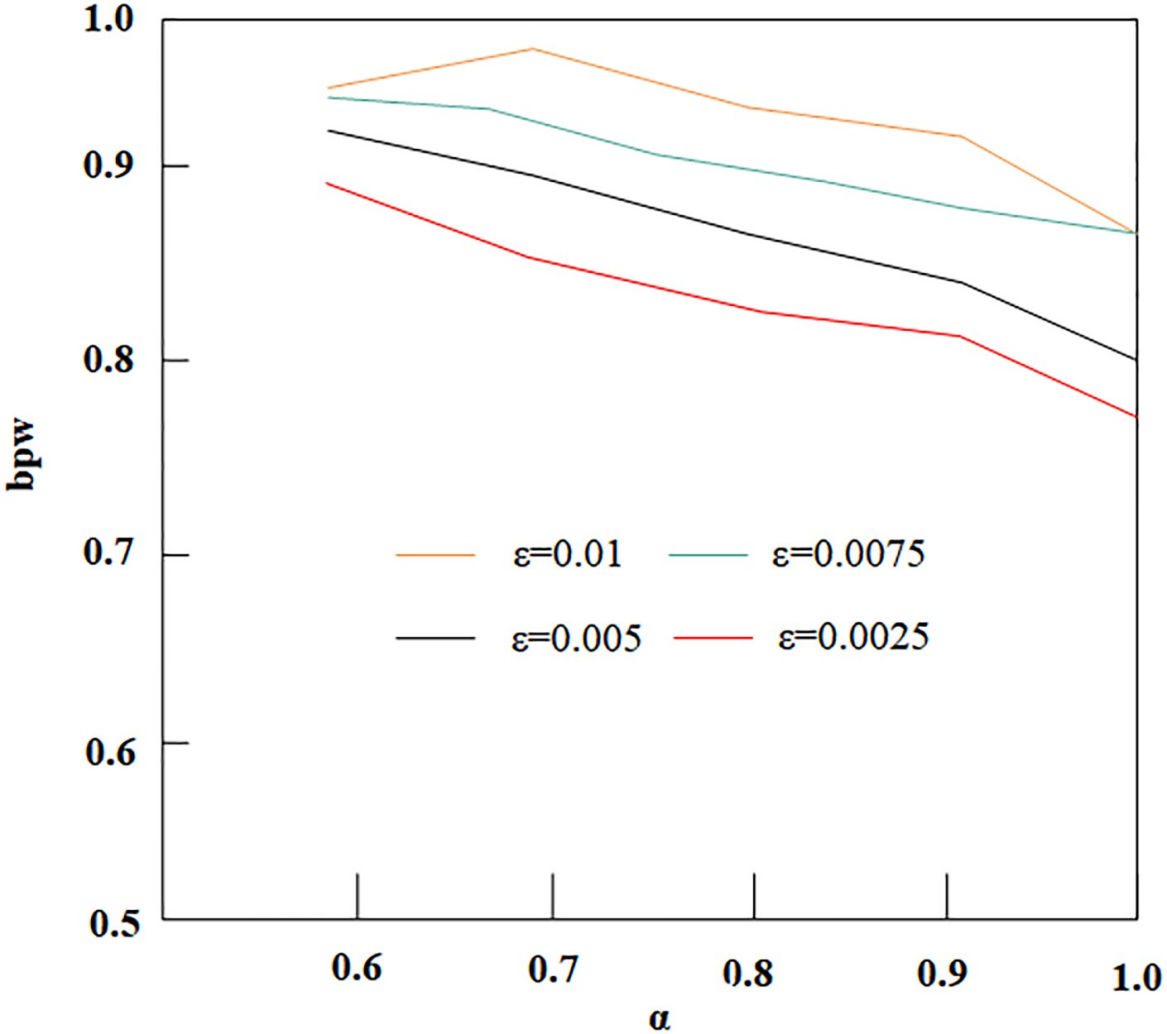
Influence of different *α* and *ε* on bpw.

As shown in Fig 7, as *α* increases, the embedding rate bpw (bits per word) of different models decreases. This is because α reflects the dependency level of the current generated word *y*_*t*_ on the source sentence. When *α* decreases from 1 to 0.6, the constraints on the current word *y*_*t*_ are reduced, which expands the selectable range of alternative set elements and ultimately increases the size of the alternative set. Thus, reducing the value of *α* can increase the model’s embedding capacity.

#### 3.3.2 The Influence of α on the PPL

**Fig 8:**
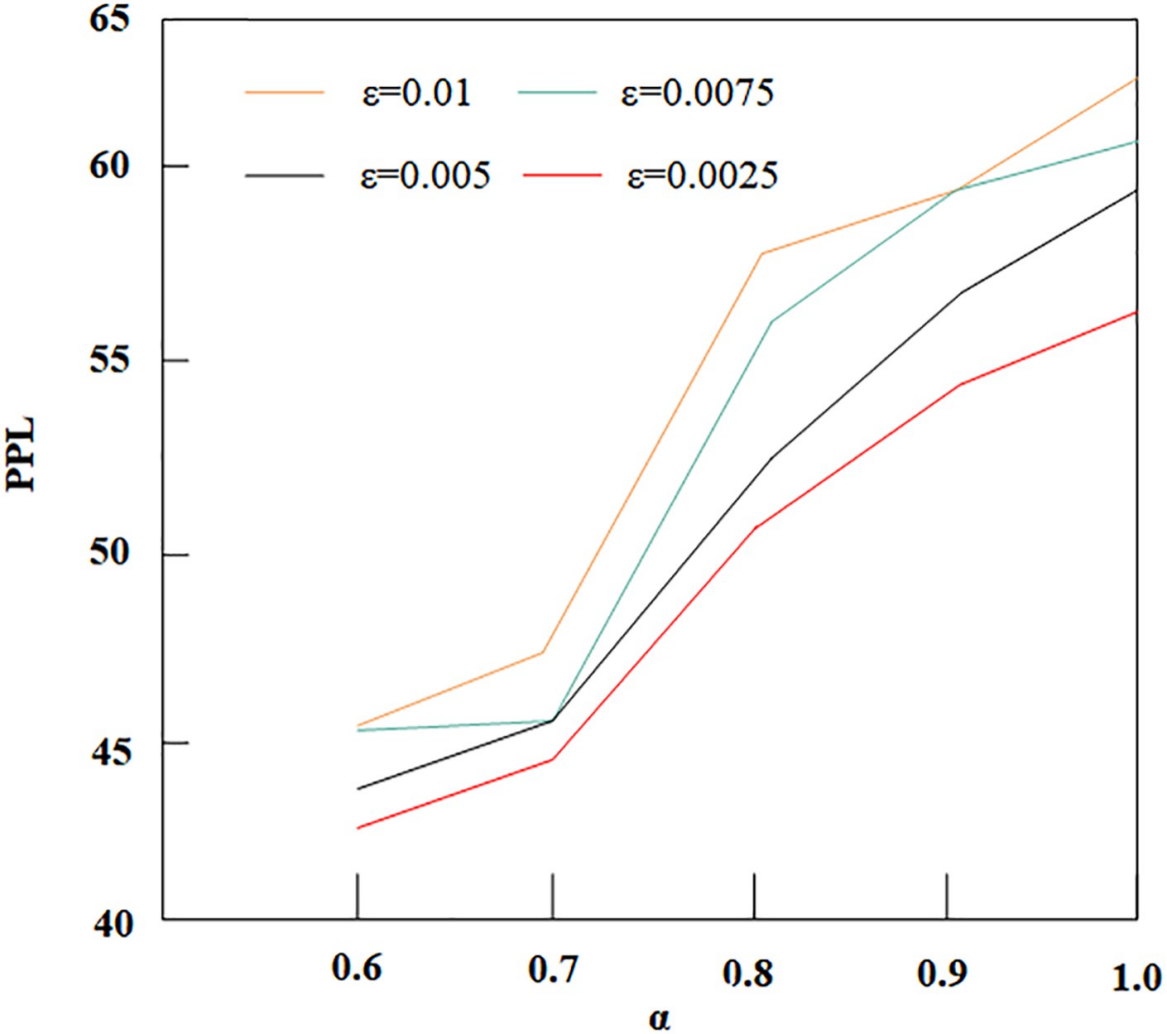
Influence of different *α* and *ε* on PPL.

Fig 8 reveals that as *α* increases, PPL also increases, which is opposite to bpw. This indicates that appropriately reducing the value of *α* not only increases the model’s embedding capacity but also reduces the complexity of the language model. This is because when the dependency of yt on the source sentence is reduced, the generation of yt relies more on the already generated parts. It is these parts that provide more useful information, making the distribution of the final generated sentences closer to the true distribution of the target text.

#### 3.3.3 The influence of α on the BLEU

**Fig 9:**
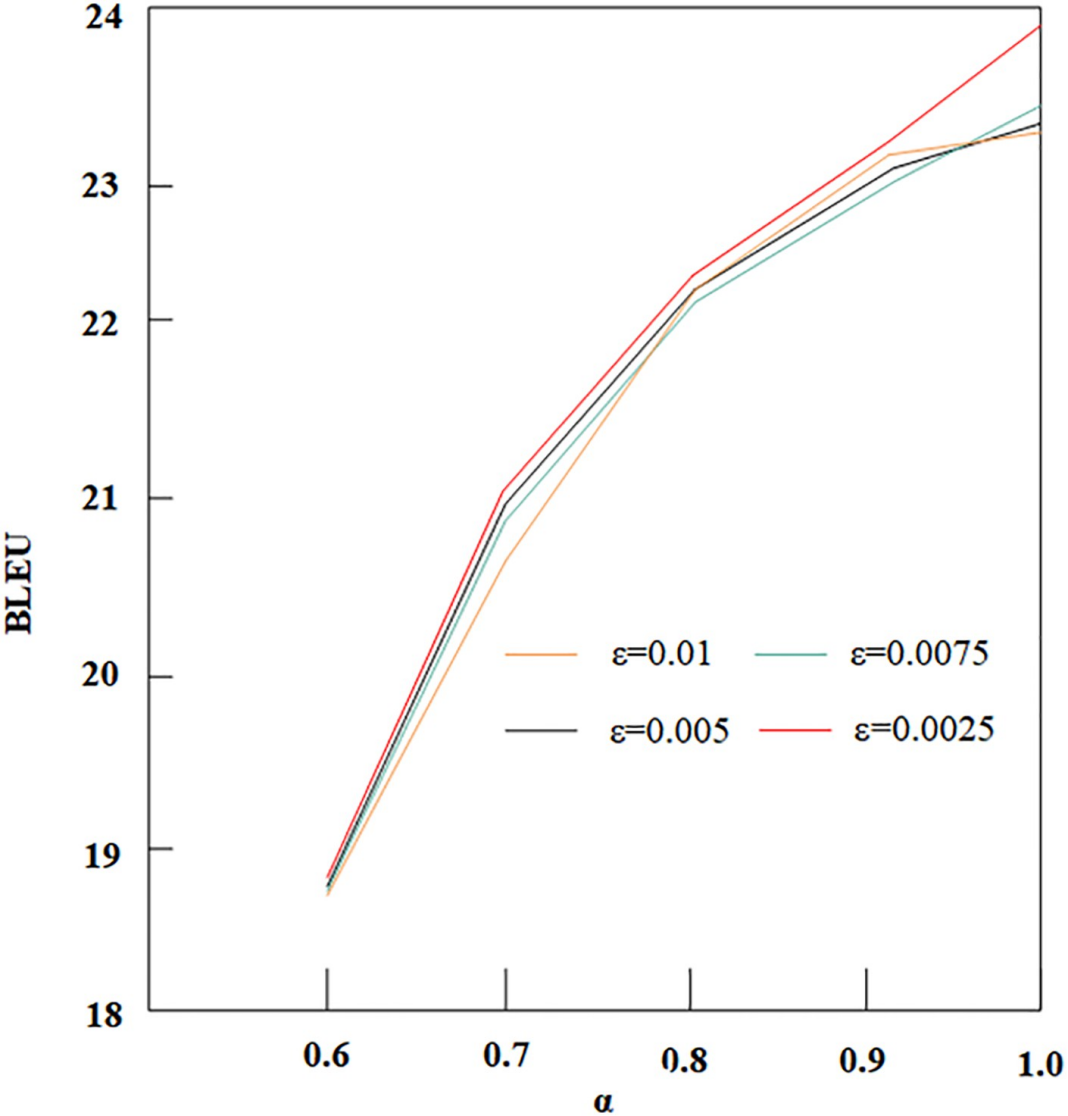
Influence of different *α* and *ε* on BLEU.

Fig 9 shows that BLEU decreases as *α* decreases, further confirming that changing the value of *α* affects the dependency of *y*_*t*_ on the source sentence.

Table 2 presents the experimental results of PPL, BLEU, and *bpw* under different variance thresholds *ε* and attention hyper-parameters *α*, with varying *bs* values.

**Table 2:** Influence of different parameters on bwp, BLEU and PPL.

(A)					
*ε*	*bs*	*α*	*bpw*	*PPL*	BLEU
0.07	2	1	0.663	55.860	23.592
0.072			0.715	57.915	23.286
0.074			0.758	59.482	23.370
0.076			0.758	62.499	23.216
0.07	4		0.778	63.507	23.516
0.08			1.059	67.658	23.202
0.09			1.120	72.800	23.316
0.1			1.174	75.913	23.050
0.045	8		0.841	69.731	23.368
0.05			0.981	73.123	23.131
0.055			1.104	79.709	22.898
0.06			1.320	81.373	22.468
(B)					
*ε*	*bs*	*α*	*bpw*	*PPL*	*BLEU*
0.07	2	1	0.770	54.399	23.207
0.072			0.815	55.437	22.962
0.074			0.843	57.985	22.900
0.076			0.866	58.031	23.074
0.07	4		1.180	63.046	23.213
0.08			1.265	63.268	22.921
0.09			1.297	68.503	23.034
0.1			1.390	73.664	22.833
0.045	8		1.243	64.391	22.970
0.05			1.395	72.113	22.669
0.055			1.457	74.640	22.699
0.06			1.650	79.044	22.236
(C)					
*ε*	*bs*	*α*	*bpw*	*PPL*	*BLEU*
0.07	2	1	0.787	50.169	22.339
0.072			0.835	52.201	22.271
0.074			0.867	55.249	22.151
0.076			0.896	57.267	22.273
0.07	4		1.199	59.267	22.245
0.08			1.301	60.397	22.144
0.09			1.332	62.409	22.143
0.1			1.446	67.547	21.813
0.045	8		1.265	62.408	22.269
0.05			1.452	71.735	21.803
0.055			1.518	72.801	21.674
0.06			1.762	73.345	21.196
(D)					
*ε*	*bs*	*α*	*bpw*	*PPL*	*BLEU*
0.07	2	1	0.824	44.105	20.883
0.072			0.883	45.148	20.815
0.074			0.924	45.167	20.751
0.076			0.949	47.144	20.589
0.07	4		1.265	52.200	20.932
0.08			1.393	52.270	20.804
0.09			1.488	55.270	20.669
0.1			1.572	62.448	20.272
0.045	8		1.352	51.329	20.810
0.05			1.604	59.586	20.516
0.055			1.803	63.176	20.095
0.06			1.988	65.112	19.777
(E)					
*ε*	*bs*	*α*	*bpw*	*PPL*	*BLEU*
0.07	2	1	0.896	42.103	18.774
0.072			0.923	43.144	18.678
0.074			0.944	45.138	18.697
0.076			0.948	45.193	18.583
0.07	4		1.398	45.177	18.700
0.08			1.553	50.258	18.515
0.09			1.678	50.327	18.108
0.1			1.775	52.443	18.063
0.045	8		1.548	44.348	18.510
0.05			1.878	46.549	18.080
0.055			2.102	50.751	18.054
0.06			2.211	55.065	17.519

From Table 2, it can be concluded that as α decreases, both PPL and BLEU decrease, while bpw increases. Moreover, increasing bs and variance threshold ε also increases the model’s embedding capacity and complexity, with BLEU decreasing.

When the embedding rate is too high, the quality of the generated carrier text may decline due to the impact on the quality of alternative set elements. For example, given the source text: *An area of approximately 330 hectares of foreshore and seabed are affected by the works as required in the gazette today April 14* (In Chinese), a high embedding rate may produce the carrier text: *The scope of the service is well affected by the three stages of the project*, *which is scheduled for today April 15 in 2004* (In English). Reducing the embedding rate results in better carrier text such as *An area of approximately 330 hectares of foreshore and seabed are affected by the works as required in the gazette today April 14* (In English).

### 3.4 Contrast experiment

This study compares three generative linguistic steganography models, including two different Markov-based steganography models [34, 35] and one RNN-based generative text steganography model, i.e., RNN-stega [22]. Specifically, literature [34] utilizes Markov models and Huffman coding to embed secret information by analyzing the statistical features of text. It first establishes a Markov model of the text and then employs Huffman coding to embed the secret information into the text, minimizing its impact. This method emphasizes the efficient concealment of information while preserving the naturalness of the text. By utilizing the Markov model, it can better maintain the statistical characteristics of the text. On the other hand, literature [35] focuses on a language model based on Markov chains, modifying the order of text to embed secret information. It utilizes the properties of Markov chains to embed information into the text and reconstructs the Markov chain during extraction to retrieve hidden information. This approach emphasizes achieving information concealment within the text by imitating the characteristics of natural text to enhance security. As for the introduction of RNN-stega, please refer to the introduction section.

We utilize two linguistic steganalysis tools, namely NFZ-WDA [36] and LS-CNN [37], to evaluate the security of the carrier texts. NFZ-WDA is a specialized steganalysis model for detecting neural network-based steganography that uses *n*-gram algorithms to identify statistical feature variations between the carrier texts and reference texts. NFZ-WDA is a specialized steganalysis model based on the observation that all authors leave distinct inherent traces of vocabulary use in their written texts, which can be recognized and used for authorship analysis. By analyzing the distribution of intrinsic words within the text using the *n*-gram algorithm, the inherent word usage style within the text can be estimated to detect statistical feature changes between the carrier text and the reference text. On the other hand, LS-CNN first uses the word embedding layer to extract the semantic and syntactic features of words, and then learns sentence features using rectangular convolutional kernels of different sizes to capture complex long-text dependencies and detect distribution differences between the carrier text and the reference text.

In this study, conventional training methods were not employed, where steganalysis tools are trained separately for different *bs*. As real-world carrier texts are often a mixture of various data, we aim to approximate real-world scenarios and train a single steganalysis model with the mixed carrier texts of different *bs*. For instance, when training LS-CNN to detect carrier texts, the training dataset contains a total of 15,000 carrier sentences generated from various payload bs, attention parameters *α*, and variance thresholds *ε*.

Furthermore, to validate the long-range semantical correlations between the generated carrier sentences, we computed the semantical distances and the average sentence lengths of the generated carrier sentences. Semantical distance is a metric used to measure the semantic similarity between texts. In steganalysis, it is often employed to compare the semantical similarity between carrier text and reference text. If the embedded secret information alters the semantical content of the text, the semantical distance may increase. As a steganalysis metric, it helps detect whether a text contains hidden information. A larger semantic distance may indicate an increased difference between texts, possibly due to steganographic operations. Average sentence length is the mean length of sentences in a text. In steganalysis, this metric is typically used to detect changes in the text. When secret information is embedded in a text, the average sentence length may change because the embedded information can lead to certain sentences becoming shorter or longer. By comparing the average sentence length between carrier and reference text, changes can be detected, hinting at potential steganographic operations in the text. The semantical distance was computed using OpenAI GPT model [38]. The steganalysis accuracy and semantical distance results for different models are shown in Table 3.

**Table 3:** Comparison of experimental results under different experimental configurations. Note: ↓ indicates that smaller values are better, and ↑ indicates that larger values are better.

Method	*ε*	*bpw*	NFZ-WDA↓	LS-CNN↓	Semantical distance↓	Average length↑	*PPL*↓	*BLEU*↑
Markov [34]	N/A	1	82.5	95.2	354.317	17	493.992	0.994
	N/A	2	80.5	94.8	355.293	17	577.628	0.731
	N/A	3	82.5	95	368.158	23	585.311	0.863
Markov [35]	N/A	1	88.5	94.9	329.113	19	294.578	1.681
	N/A	2	84.5	96.6	360.997	20	486.043	0.973
	N/A	3	82	96.8	373.532	25	531.080	0.607
RNN-stega [22]	N/A	1	76.5	80.5	280.612	26	44.080	10.362
	N/A	2	73	88	324.685	28	67.915	8.041
	N/A	3	64.5	89.8	331.454	31	136.542	5.679
NMT-stega	0.045	0.841	**53**	56.5	**87.916**	46	69.731	**23.368**
	0.05	0.981	53.5	58.2	89.941	46	73.123	23.131
	0.055	1.104	53.5	59	93.218	46	79.709	22.898
	0.06	1.320	56	61.5	94.301	46	81.373	22.468
	0.045	1.548	55.5	**53.4**	92.019	**47**	**44.348**	18.510
	0.05	1.878	52.5	55	96.428	**47**	46.549	18.08
	0.055	2.102	57.5	59.7	106.440	**47**	50.751	18.054
	0.06	2.211	55.5	65.5	113.629	**47**	55.065	17.519

Table 3 indicates that the proposed model outperforms the other three models in terms of anti-steganography attacks and preserving semantical relationships between sentences. Compared to RNN-stega, the detection accuracy of NFZ-WDA and LS-CNN on the carrier texts generated by our model reduced by 21.5% and 28.3%, respectively, at the same embedding rate. This indicates that our model exhibits higher security and better resistance against malicious attacks. The proposed NMT-stega model generates carrier text with a maximum average sentence length of 47, whereas the previous methods had a maximum sentence length of only 31. This indicates that our method introduces more significant changes in sentence structure, resulting in longer sentences. Additionally, the minimum semantical distance between sentences reached 87.916, which is significantly lower than the minimum semantical distance of the previous methods. This suggests that the generated carrier texts exhibit higher semantical similarity between sentences, making them more natural and preserving more of the original semantical information compared to the previous methods. In summary, our method can effectively maintain semantical relationships between long sentences, even when generating lengthy text.

Moreover, combining Tables 2 and 3, it can be seen that our method achieved the best PPL of 42.103 and a BLEU score of 23.592. Compared to the previous best method, RNN-stega, these represent improvements of 1.977 and 13.23, respectively. In conclusion, the proposed method excels at the machine translation task while effectively resisting steganalysis attacks.

### 3.5 Efficiency analysis

The storage space for this method primarily comes from the encoder-decoder structure, occupying approximately 182MB of memory. Other components, such as the alternative set, require about 1.4MB of space, while the memory usage for intermediate variables is almost negligible. During the training phase, the neural network consumes approximately 7.3 hours. On average, during the inference phase, the time required to complete one pass of the neural network is around 268ms. Constructing the binary tree takes about 17ms, resulting in an estimated time of about 311ms for embedding information in a single sentence and approximately 304ms for extracting information from a single sentence. The reason that the secret information embedding algorithm consumes more time compared to the extraction algorithm is that it needs to generate the carrier text word by word. In contrast, the extraction algorithm primarily involves comparing the received carrier sentence with alternative words for information extraction, which tends to be a faster process as it doesn’t entail text generation but only involves comparison and matching operations. Considering the superiority of this method in the anti-steganography attacks and maintaining inter-sentence semantics, these results demonstrate an acceptable performance level in the sensitive field of linguistic steganography.

## 4. Conclusion

In this paper, we combine NMT model with linguistic steganography model, resulting in the NMT-stega model. Firstly, we adopt an attention-fused Encoder-Decoder architecture to dynamically embed secret information during the text generation process. As the generated text needs to maintain semantical connections with distant words, the pick of each target text takes into account both the source text and the previously generated target words. Secondly, the proposed word pick policy based on probability variance allows for varying the number of words in the alternative set at each time step. This adaptive secret information embedding policy adjusts the embedding capacity based on the current conditional probability distribution. Finally, we analyze the impact of the proposed hyper-parameter based on prior knowledge on the embedding rate and text generation quality of the model.

The achievements of this study provide researchers with intriguing directions and visions for future work. Here is a discussion:

1. Enhancing NMT-stega Model Performance: Future work can focus on improving the performance of the NMT-stega model to increase the embedding rate and text generation quality. This can be achieved through more complex encoder-decoder architectures, refined attention mechanisms, and advanced word selection strategies.
2. Exploring Diverse Applications: The success of the NMT-stega model’s application can be extended to various fields. Researchers can explore how this technology can be used to improve steganalysis, enhance information security, or advance other applications of steganography.
3. Increasing Model Robustness: Robustness is a key concern in the field of linguistic steganography. Future research can concentrate on developing more robust NMT-stega models to withstand various text processing and analysis attacks.
4. Advancements in Steganalysis and Detection: As linguistic steganography continues to evolve, steganalysis and detection techniques need to keep pace. Researchers can explore new methods and algorithms to improve the detection capabilities of linguistic steganography.

## Supporting information

S1 File (RAR)
